# Supplementary material for: Making silver a stronger n-dopant than cesium via in situ coordination reaction for organic electronics
Source: Nat Commun. 2019 Feb 20;10:866. doi: 10.1038/s41467-019-08821-x (PMC6382909; doi:10.1038/s41467-019-08821-x)
Supplement: Supplementary file 1 — Supplementary Information [file 41467_2019_8821_MOESM1_ESM.pdf]

Supplementary information

Making Silver a Stronger N-dopant than Cesium via *in situ* Coordination Reaction for Organic Electronics

Zhengyang Bin[1,2], Guifang Dong[1], Pengcheng Wei[1], Ziyang Liu[1], Dongdong Zhang[1], Rongchuan Su[2], Yong Qiu[1], Lian Duan[1,3,]*

[1] *Key Lab of Organic Optoelectronics, Department of Chemistry, Tsinghua University, Beijing, 100084, China*

[2] *College of Chemistry, Sichuan University, Chengdu, Sichuan, 610064, China*

[3] *Center for Flexible Electronics Technology, Tsinghua University, Beijing, 100084, China*

**Corresponding author: duanl@mail.tsinghua.edu.cn*

Supplementary Figures

a

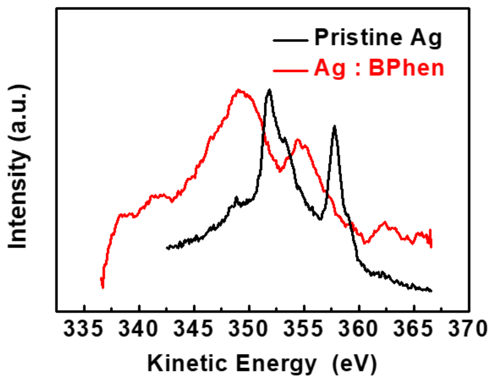

b

Sample		Ag 3d Binding Energy (eV)	Ag MNN Kinetic Energy (eV)
Reference	Ag	368.2	357.9
	Ag_2SO_4	368.3	354.7
	Ag_2O	368.4	350.6
	$AgOOCCF_3$	368.8	355.1
Experiment	Ag	368.3	357.8
	Ag:BPhen	368.8	354.3

Supplementary Figure 1 l (a) AES spectra of pristine Ag and Ag-doped BPhen films. (b) The binding energy of Ag 3d and the kinetic energy of Ag MNN for different Ag samples.

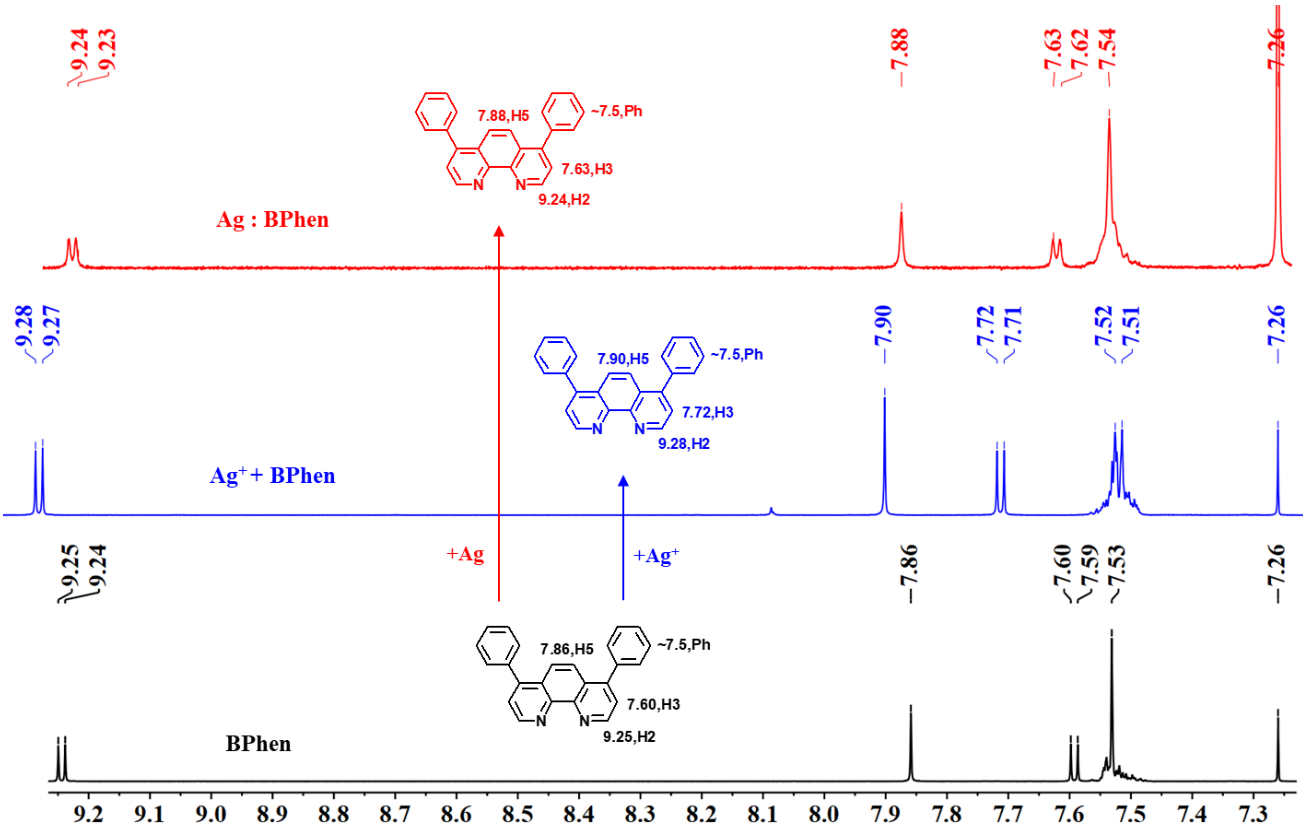

Supplementary Figure 2 l The comparison of chemical shifts for pristine BPhen, Ag^{+}-doped BPhen and Ag-doped BPhen. (For pristine BPhen (black) and Ag:BPhen (red), their films were firstly prepared by vacuum deposition, following by $CDCl_3$ dissolving and nuclear magnetic resonance (NMR) measurement. And for Ag^{+}+BPhen (blue), $AgNO_3$ was added to $CDCl_3$ solution of BPhen for NMR measurement.)

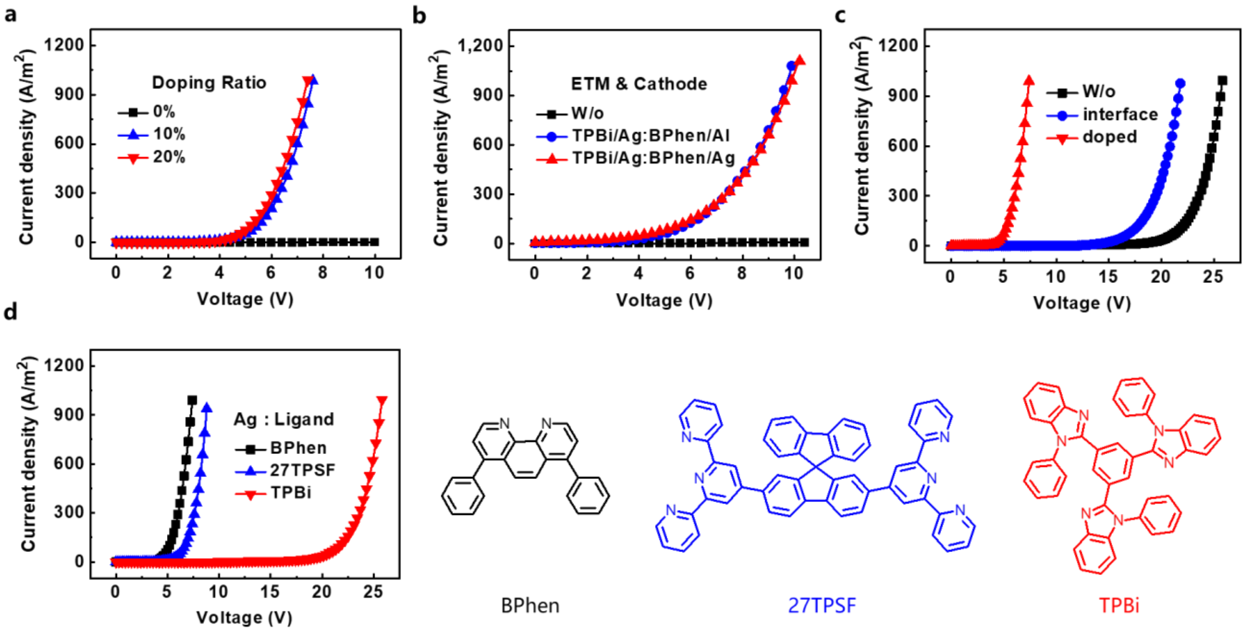

Supplementary Figure 3 I Current density-voltage characteristics of electron-only devices (EODs) (a) with different doping ratios (ITO/ BPhen (100 nm)/ Ag-doped BPhen = *x* % (5 nm)/ Al), (b) with different electron transport layers and cathodes (ITO/ TPBi (100 nm)/ Ag-doped BPhen = 20 % (5 nm)/ Al or Ag (10 nm)/Al), (c) with different electron injection layers (W/o: ITO/ BPhen (100 nm)/ Al, interface: ITO/ BPhen (100 nm)/ Ag (10 nm)/ Al and doped: ITO/ BPhen (100 nm)/ Ag-doped BPhen = *x* % (5 nm)/ Al) and (d) with different organic ligands and the molecular structures (ITO/ BPhen (100 nm)/ Ag-doped ligand = 20 % (5 nm)/ Al).

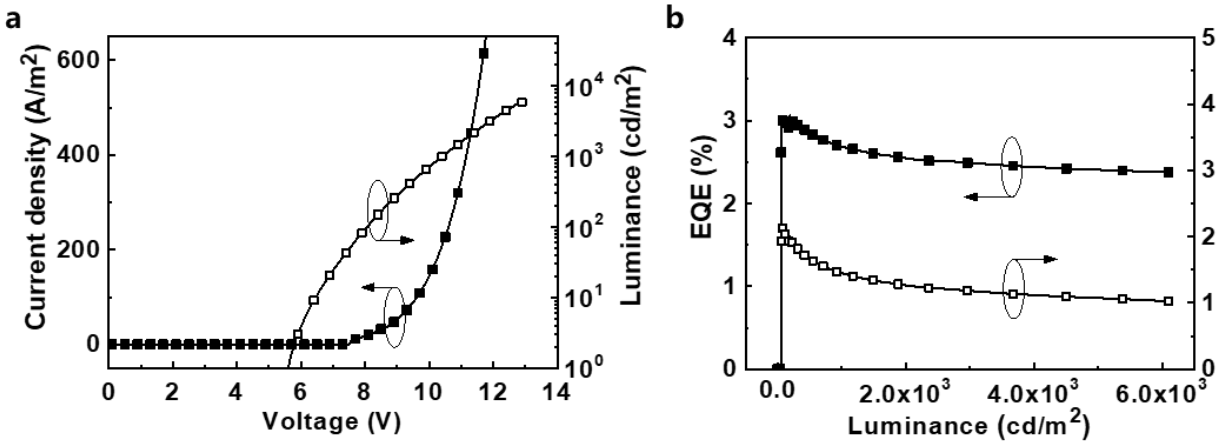

Supplementary Figure 4 l (a) The current density-luminance-voltage and (b) the current efficiency-power efficiency-luminance of OLED device without using any n-dopant for electron injection.

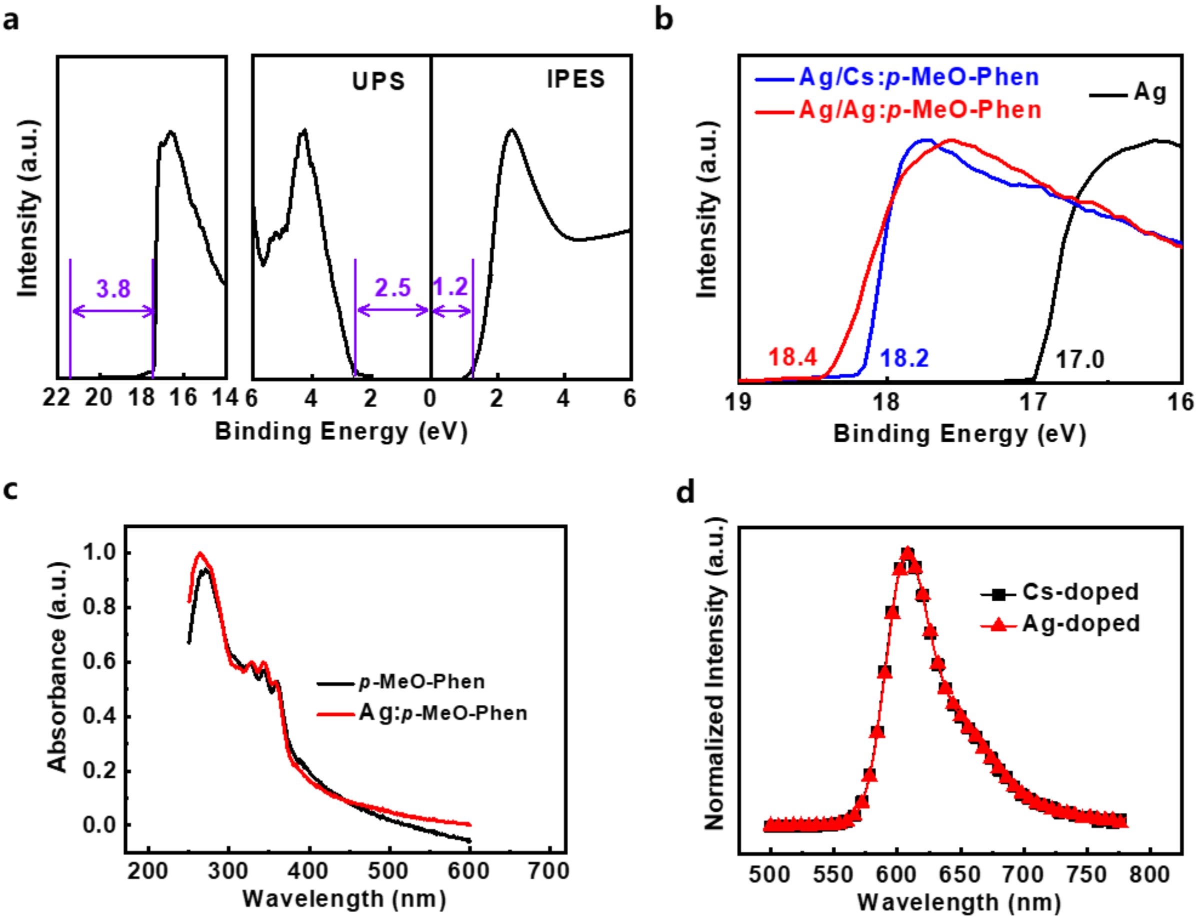

Supplementary Figure 5 | (a) The UPS and LEIPS spectra of pristine *p*-MeO-Phen film. (b) Photoemission cutoff obtained via UPS spectra. (c) The absorption spectra of pristine and Ag-doped *p*-MeO-Phen films. (d) The normalized EL curves of OLED devices using Cs and Ag as n-dopants for comparison.

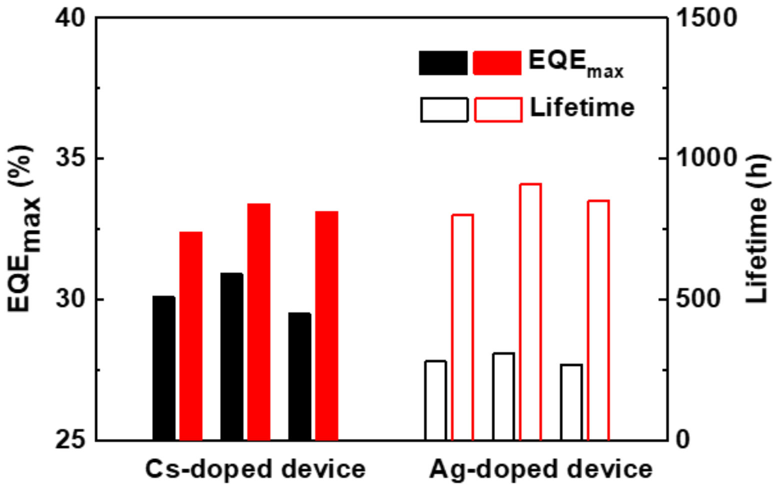

Supplementary Figure 6 l The histograms of EQEs$_{max}$ and lifetimes measured from different devices based on Cs-doped and Ag-doped EILs.

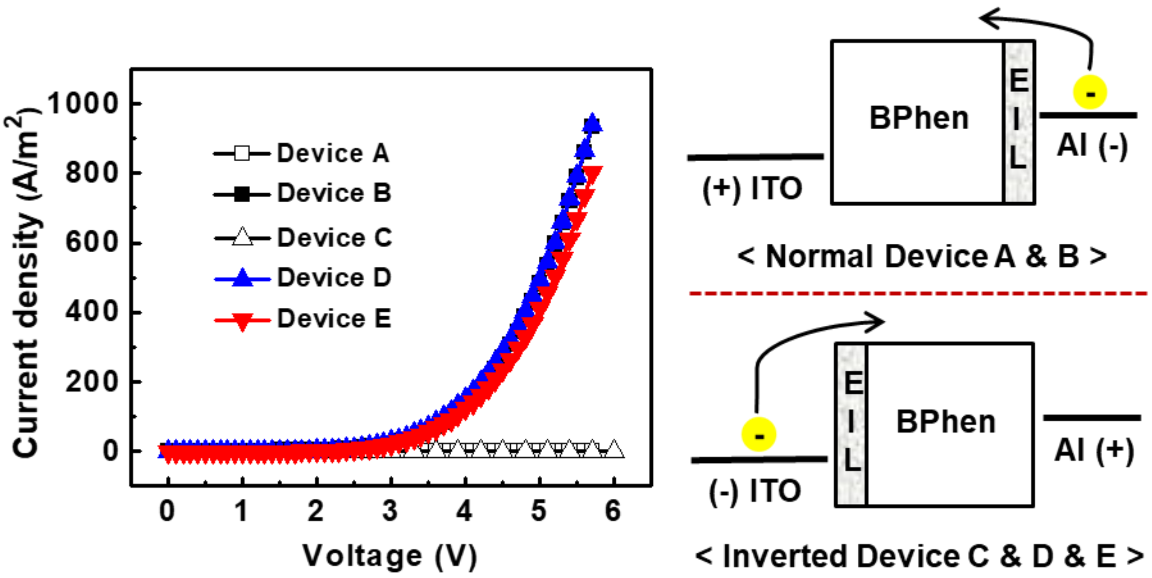

Supplementary Figure 7 | Current density-voltage characteristics of EODs with Ag-doped *p*-MeO-Phen film depositing onto different kinds of electrodes (Metal: Al, Metal oxide: ITO, or Organic: HAT-CN). Device structures are as follows:

Device A: (+) ITO/ BPhen (100 nm)/ *p*-MeO-Phen (5 nm)/ Al (-)

Device B: (+) ITO/ BPhen (100 nm)/ Ag-doped *p*-MeO-Phen (5 nm)/ Al (-)

Device C: (-) ITO/ *p*-MeO-Phen (5 nm)/ BPhen (100 nm)/ Al (+)

Device D: (-) ITO/ Ag-doped *p*-MeO-Phen (5 nm)/ BPhen (100 nm)/ Al (+)

Device E: (-) ITO/ HAT-CN (10 nm)/Ag-doped *p*-MeO-Phen (5 nm)/ BPhen (100 nm)/ Al (+)

Supplementary Notes

Supplementary Note 1 | Synthetic routes for 2,9-dimethoxy-4,7-diphenyl-1,10-phenanthroline (*o*-MeO-BPhen)

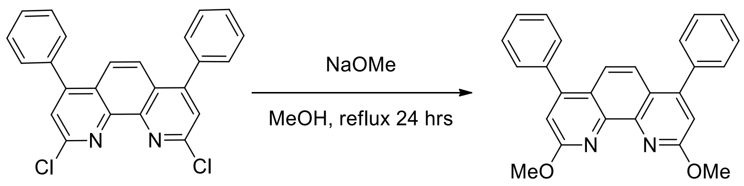

30 g (556 mmol, 14.8 eq) sodium methoxide and methanol were added in a 1000 mL three-necked flask equipped with a magnetic stirrer, 2,9-dichloro-4,7-diphenyl-1,10-phenanthroline 15 g (37.5 mmol, 1 eq) under nitrogen protection. The mixture was heated and reflux for 24 hours. Then the reaction was cooled to room temperature and the solvent was dried under reduced pressure. 300 mL of dichloromethane and 100 mL of pure water were added and stirred at room temperature until the system became clear. The aqueous phase was extracted with dichloromethane (100*2 mL). The organic phases were combined, dried over anhydrous sodium sulfate, and suction filtered. The filtrate was directly filtered through a short silica gel column (80 g, 200-300 mesh silica gel) under reduced pressure and the column was eluted with PE : EtOAc = 300 : 100 mL. The solvent was dried under reduced pressure to give about a tan solid product. This solid was recrystallized from toluene / methanol = 30 mL / 90 mL to give an off-white powdery solid. The final *o*-MeO-BPhen product was further purified by sublimation under high vacuum. The yield was 45%.

^{1}H NMR (600 MHz, $CDCl_3$) δ/ppm: 7.61 (s, 2H), 7.5 (m, 5H), 7.06 (s, 2H), 4.34 (d, 6H).

^{13}C NMR (151 MHz, $CDCl_3$) δ/ppm: 162.30, 151.65, 138.18, 129.70, 129.51, 128.43, 123.73, 113.66, 113.30, 100.00, 53.41.

ESI-MS m/z: 393.16 $[M+H]^+$.

Supplementary Note 2 l Synthetic routes for 4,7-dimethoxy-1,10-phenanthroline (*p*-MeO-Phen)

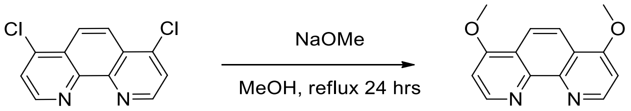

30 g (556 mmol, 14.8 eq) sodium methoxide and methanol were added in a 1000 mL three-necked flask equipped with a magnetic stirrer, 4,7-dichloro-1,10-phenanthroline 9.3 g (37.5 mmol, 1 eq) under nitrogen protection. The mixture was heated and reflux for 24 hours. Then the reaction was cooled to room temperature and the solvent was dried under reduced pressure. 300 mL of dichloromethane and 100 mL of pure water were added and stirred at room temperature until the system became clear. The aqueous phase was extracted with dichloromethane (100*2 mL). The organic phases were combined, dried over anhydrous sodium sulfate, and suction filtered. The filtrate was directly filtered through a short silica gel column (80 g, 200-300 mesh silica gel) under reduced pressure and the column was eluted with PE : EtOAc = 300 : 100 mL. The solvent was dried under reduced pressure to give about a tan solid product. This solid was recrystallized from toluene / methanol= 30 mL / 90 mL to give an off-white powdery solid. The final *p*-MeO-Phen product was further purified by sublimation under high vacuum. The yield was 50%.

^{1}H NMR (600 MHz, $CDCl_3$) δ/ppm: 8.98 (d, 2H), 8.14 (s, 2H), 6.95 (d, 2H), 4.04 (s, 6H).

^{13}C NMR (151 MHz, $CDCl_3$) δ/ppm: 162.30, 151.21, 146.74, 120.97, 119.02, 102.81, 55.91.

ESI-MS m/z: 241.10 $[M+H]^+$.
